# Associations of the systemic immune-inflammation index and systemic inflammatory response index with chronic obstructive pulmonary disease: a systematic review and meta-analysis

**DOI:** 10.3389/fmed.2026.1853538

**Published:** 2026-06-26

**Authors:** Li-fang Yang, Zheng Yang

**Affiliations:** 1 Obstetrics and Gynecology, Qujiang District Maternal and Child Health Care Hospital, Quzhou, Zhejiang, China; 2 Department of Infectious Diseases, The Second People’s Hospital of Quzhou, Quzhou, Zhejiang, China

**Keywords:** COPD, COPD presence and prognosis, Meta-analysis, SII, SIRI

## Abstract

**Background:**

Recent evidence suggests that Systemic Immune-Inflammation Index (SII) and Systemic Inflammatory Response Index (SIRI) are associated with the presence and prognosis of patients with chronic obstructive pulmonary disease (COPD). However, existing research results remain controversial.

**Methods:**

PubMed, Embase, Web of Science, and the Cochrane Library were systematically searched from inception to March 23, 2026. Observational studies investigating the associations of SII or SIRI with COPD risk, all-cause mortality (ACM), or respiratory failure (RF) were included. Data were analyzed using Review Manager 5.4.1 and Stata 15.1. Heterogeneity was assessed using Cochran’s *Q* test and the *I*^2^ statistic, with subgroup analyses conducted when substantial heterogeneity was detected. Sensitivity analyses were performed using a leave-one-out approach to evaluate the robustness of the findings. Publication bias was assessed through funnel plots and Egger’s regression test. A two-sided *p <* 0.05 was considered statistically significant.

**Results:**

Eleven studies involving 59,621 participants were included. Elevated SII was significantly associated with the higher prevalence of COPD (OR = 1.22, 95% CI: 1.06–1.41), higher ACM (OR = 1.20, 95% CI: 1.09–1.36), and an higher risk of RF (OR = 1.61, 95% CI: 1.28–2.02). In contrast, no significant association was observed between SIRI and COPD risk (OR = 1.14, 95% CI: 0.90–1.45). Subgroup analyses indicated that the association between SII and COPD risk was not statistically significant in cohort studies, populations aged ≥65, studies conducted in Europe, and patients with acute exacerbations of COPD (AECOPD). Sensitivity analyses confirmed the stability of the pooled estimates, and no significant publication bias was detected by funnel plots or Egger’s test.

**Conclusion:**

Compared with SIRI, SII is an easily measurable and promising biomarker. Existing evidence confirms its association with COPD prevalence, ACM, and RF. Further prospective studies shall establish standardized cutoff values and validate their clinical utility across diverse populations.

## 1 Introduction

Chronic obstructive pulmonary disease (COPD) is a prevalent chronic respiratory disorder affecting approximately 330 million individuals worldwide and is currently the third leading cause of death globally, following cardiovascular disease (CVD) and stroke (1). Its prevalence increases with age and is most commonly observed among individuals aged over 40. Although COPD remains more prevalent in men, its prevalence among women has steadily increased in recent decades (2). Smoking is the most important risk factor for COPD, while air pollution and occupational dust exposure also contribute substantially to disease development (3, 4). Current management strategies for COPD include both pharmacological and non-pharmacological interventions (5). Pharmacological treatments mainly consist of bronchodilators, such as long-acting *β*₂-agonists and muscarinic antagonists, as well as inhaled corticosteroids, which aim to alleviate symptoms, improve lung function, and reduce the frequency of acute exacerbations (6). Non-pharmacological approaches include long-term oxygen therapy, pulmonary rehabilitation, vaccination, and smoking cessation support (7, 8). Despite these therapeutic advances, COPD remains an incurable disease, and progressive decline in lung function is largely unavoidable (9). The prognosis of COPD is closely associated with disease severity, acute exacerbations, and the presence of comorbidities (10, 11). Patients frequently present with comorbid conditions, including CVD and diabetes mellitus, which substantially increase mortality risk (12).

COPD is fundamentally characterized as a chronic inflammatory disease (13, 14). Persistent airway inflammation, primarily triggered by prolonged exposure to noxious stimuli such as tobacco smoke and environmental pollutants, represents the central pathogenic mechanism underlying disease initiation and progression (15, 16). Inflammatory cells, including neutrophils, macrophages, and T lymphocytes, particularly CD8^+^ T cells, accumulate within the airways and release a variety of inflammatory mediators, such as interleukin-6 (IL-6), tumor necrosis factor-*α* (TNF-α), neutrophil elastase, and matrix metalloproteinases (MMPs). These mediators contribute to airway wall destruction, excessive mucus production, alveolar structural damage (emphysema), and airway fibrosis (17, 18). Notably, inflammation in COPD is not restricted to the respiratory system but may also become systemic, affecting skeletal muscle, the cardiovascular system, and other organs, thereby aggravating comorbid conditions (19, 20). The Systemic Immune-Inflammation Index (SII), defined as platelet count (PC) × neutrophil count(NC)/lymphocyte count (LC), and the Systemic Inflammatory Response Index (SIRI), defined as NC × monocyte count/LC, have emerged as novel biomarkers reflecting systemic inflammatory status. Accumulating evidence indicates that elevated SII and SIRI levels are associated with an increased COPD risk and poorer clinical outcomes. For example, Zhang et al. (21), using data from the MIMIC-IV database that included 1,653 COPD patients, demonstrated that SII effectively predicted in-hospital respiratory failure (RF) and mortality. Similarly, Ye et al. (22), based on National Health and Nutrition Examination Survey (NHANES) data from 16,636 adults aged ≥40 with a median follow-up of 146 months, reported that participants in the high-SII group had a 44.9% higher risk of COPD and a 34.1% increased risk of all-cause mortality (ACM). Elevated SII and SIRI levels have also been associated with ACM and severe complications, including RF, among patients with COPD. By integrating information from multiple inflammatory cell lineages, these indices may provide a more comprehensive assessment of systemic inflammation than single-cell-derived markers such as the neutrophil-to-lymphocyte ratio (NLR), while remaining simple, readily available, and cost-effective tools for early identification, risk stratification, and prognostic assessment.

To date, although the diagnostic and prognostic significance of SII and SIRI in COPD has been investigated in several individual studies, no meta-analysis has comprehensively synthesized the available evidence. Therefore, the study aimed to systematically evaluate the associations of SII and SIRI with COPD prevalence and prognosis, thereby laying a more rigorous theoretical basis for the clinical intervention and prognostic evaluation of COPD.

## 2 Methods

### 2.1 Literature search

This study was conducted in accordance with the Preferred Reporting Items for Systematic Reviews and Meta-Analyses (PRISMA) 2020 statement (23). The study protocol was prospectively registered in PROSPERO (Registration No. CRD42024581818). Two investigators (YZ and YLF) jointly developed the search strategy. PubMed, Embase, Web of Science, and the Cochrane Library were systematically searched from database inception to March 23, 2026. The search strategy combined Medical Subject Headings (MeSH) terms and free-text keywords, including “Chronic Obstructive Pulmonary Disease,” “COPD,” “Chronic Obstructive Lung Disease,” “COAD,” “Chronic Obstructive Airway Disease,” “Chronic Airflow Obstruction(s),” “systemic immune-inflammation index,” “SII,” “systemic inflammation response index,” and “SIRI.” The detailed search strategy is presented in Supplementary Table S1.

### 2.2 Eligibility criteria

Inclusion criteria were: (1) studies on COPD patients and/or healthy control individuals; (2) studies investigating the association of SII and/or SIRI with COPD prevalence, ACM, or RF; (3) studies reporting, or providing sufficient data to calculate, odds ratios (ORs) with 95% confidence intervals (CIs); (4) studies including both high and low SII or SIRI groups based on predefined cutoff values; and (5) studies published in full text.

Exclusion criteria were: (1) reviews, commentaries, conference abstracts, case reports, and letters; (2) studies lacking sufficient data to estimate ORs and 95% CIs; (3) studies not reporting survival-related outcomes; and (4) studies with duplicated or overlapping data.

Two investigators (YZ and YLF) independently screened titles and abstracts, followed by full-text review. Any discrepancies were resolved through discussion and consensus. For studies derived from NHANES, participant enrollment periods were carefully examined. Although these studies originated from the same database, they included different survey cycles, eligibility criteria, and analytical variables; therefore, they were treated as independent datasets in the pooled analyses.

### 2.3 Data extraction

Data extraction was independently performed by YLF and YZ, with disagreements resolved through discussion. The following information was collected: first author, publication year, sample size, participant age, study duration, treatment strategy, timing of biomarker measurement, country, study design, cutoff values, follow-up duration, and multivariable-adjusted ORs with corresponding 95% CIs for the presence of COPD, ACM, and RF.

### 2.4 Quality assessment

The study quality was examined via the Newcastle-Ottawa Scale (NOS) (24), which assesses selection, comparability, and outcome, with a maximum score of 9. Scores of 7–9 indicate high quality (24, 25).

### 2.5 Statistical analysis

All statistical analyses were performed using Review Manager version 5.4.1. Pooled ORs and 95% CIs were calculated to estimate effect sizes. Between-study heterogeneity was assessed using Cochran’s *Q* test and the I^2^ statistic (26). Significant heterogeneity was indicated by *p* < 0.10 or *I*^2^ > 50%. Even for outcomes with low heterogeneity, random-effects models (REMs) were consistently applied to provide more conservative effect estimates and to account for potential subtle variations between studies. The possible origins of heterogeneity were examined via subgroup analyses. How individual studies influence the pooled estimates was explored utilizing sensitivity analyses. Publication bias was examined via funnel plots and Egger’s regression test (27) using Stata 15.1 (StataCorp, College Station, TX, United States). *p* < 0.05 signified statistical significance.

## 3 Results

### 3.1 Literature search results and study characteristics

**Figure 1:**
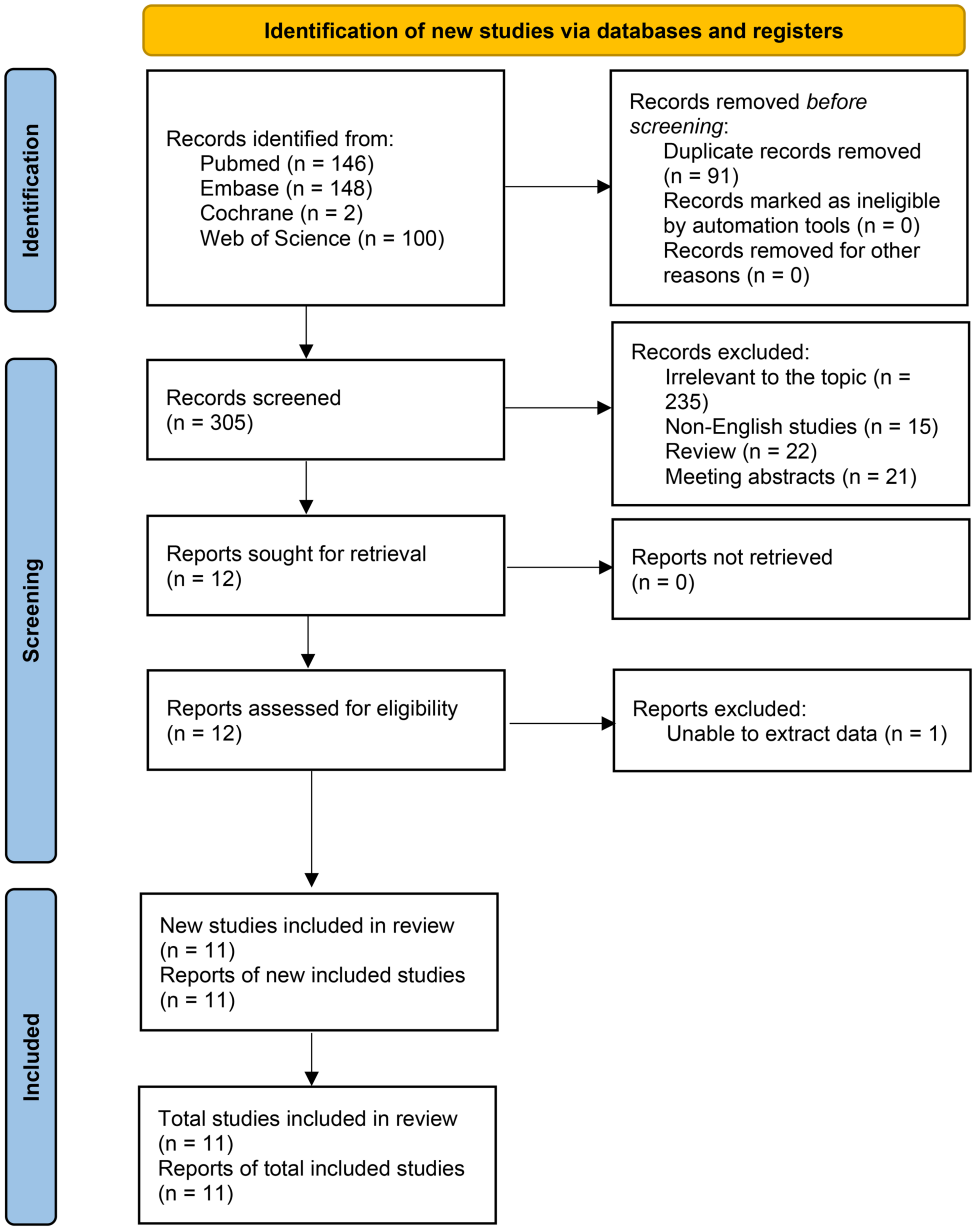
Study retrieval and selection.

A systematic search of PubMed, Embase, the Cochrane Library, and Web of Science initially identified 396 records (146 from PubMed, 148 from Embase, 2 from the Cochrane Library, and 100 from Web of Science). After removing 91 duplicate records, 305 studies underwent title and abstract screening. Subsequently, 235 irrelevant articles, 15 non-English publications, 22 reviews, and 21 conference abstracts were excluded, leaving 12 articles for full-text assessment. All full texts were successfully retrieved. Following full-text review, one study was excluded because extractable data were unavailable. Ultimately, 11 studies (21, 22, 28–36) on 59,621 individuals were encompassed (Figure 1). Because some studies contained multiple parallel datasets, 25 comparison groups were extracted from the 11 eligible studies.

Among the included studies, one was conducted in Iran, one in the Netherlands, one in Sweden, three in China, and five in the United States. The pooled dataset comprised 13 case–control comparisons, 10 retrospective comparisons, and 2 prospective comparisons. All studies were published in English between 1999 and 2024. Participants were categorized either into high versus low SII/SIRI groups according to predefined cutoff values or into COPD and non-COPD groups. Regarding inflammatory biomarkers, all 11 studies evaluated SII, whereas 4 studies assessed SIRI. Specifically, 17 comparisons examined the association between SII and COPD prevalence, 7 investigated the association between SII and ACM, 3 evaluated the association between SII and RF among COPD patients, and 4 assessed the association between SIRI and COPD prevalence. Detailed study characteristics are summarized in Table 1.

**Table 1:** Characteristics and quality assessment of included cohort studies.

Author	Study period	Region	Study design	Population	No. of patients	Gender		Mean/median Age	Mean/median BMI	Index	Cut-off	NOS score
						Male	Female					
Ye 2023 (22)	1999–2010	American	Case–control	COPD patients and healthy individuals	16,636	8,325	8,311	56.623	28.858	SII	NA	8
Ye 2023 (22)	1999–2010	American	Case–control	COPD patients and healthy individuals	16,636	8,325	8,311	56.623	28.858	SII	NA	8
Hu 2024 (29)	2014–2022	China	Case–control	TOPD patients/patients with TB	737	498	239	42.673	NA	SII	NA	8
Hu 2024 (29)	2014–2022	China	Case–control	TOPD patients/patients with TB	737	498	239	42.673	NA	SIRI	NA	8
Hosseninia 2023 (30)	2020.8–2020.12	Iran	Retrospective cohort	COPD patients hospitalized with COVID-19	169	98	71	69.27	NA	SII	939	8
Hosseninia 2023 (30)	2020.8–2020.12	Iran	Retrospective cohort	COPD patients hospitalized with COVID-19	169	98	71	69.27	NA	SIRI	1.29	8
Zuo 2019 (31)	2017–2019	China	Retrospective cohort	AECOPD/PH	185	141	44	71.18	23.16	SII	1,012	8
Zhang 2024 (49)	2008–2019	American	Retrospective cohort	COPD	1,653	879	774	71	27.4	SII	634.51	7
Zhang 2024 (49)	2008–2019	American	Retrospective cohort	COPD	1,653	879	774	71	27.4	SII	1302.17	7
Zhang 2024 (49)	2008–2019	American	Retrospective cohort	COPD	1,653	879	774	71	27.4	SII	2821.88	7
Ellingsen 2024 (32)	2014.9–2016.9	Swedish	Retrospective cohort	COPD patients	571	237	334	69	26	SII	856	8
Ellingsen 2024 (32)	2014.9–2016.9	Swedish	Retrospective cohort	COPD patients	571	237	334	69	26	SII	856	8
Ellingsen 2024 (32)	2014.9–2016.9	Swedish	Retrospective cohort	COPD patients	571	237	334	69	26	SIRI	2.024	8
Ellingsen 2024b (32)	2014.9–2016.9	Swedish	Retrospective cohort	COPD patients	571	237	334	69	26	SIRI	2.024	8
Xu 2023 (33)	2013–2020	American	Case–control	COPD aged ≥40 years	10,364	5,073	5,291	58.1	NA	SII	NA	8
Xu 2023 (33)	2013–2020	American	Case–control	COPD aged ≥40 years	10,364	5,073	5,291	58.1	NA	SII	NA	8
Song 2024 (34)	2005–2018	American	Case–control	COPD patients	2,082	1,168	914	60	NA	SII	2.74	8
Du 2024 (35)	2007–2018	American	Case–control	COPD patients	23,875	11,675	12,120	68.5	NA	SII	NA	8
Du 2024 (35)	2007–2018	American	Case–control	COPD patients	23,875			68.5	NA	SII	NA	8
Du 2024 (35)	2007–2018	American	Case–control	COPD patients	23,875			68.5	NA	SII	NA	8
Benz 2021 (28)	2009–2014	Rotterdam	Case–control	No COPD or asthma, and no sarcopenia	3,074	1,313	1761	67.2	27.5	SII	NA	9
Benz 2021 (28)	2009–2014	Rotterdam	Case–control	COPD without sarcopenia	589	341	248	69.1	26.4	SII	NA	9
Benz 2021 (28)	2009–2014	Rotterdam	Case–control	COPD with sarcopenia	92	57	35	78.7	26.4	SII	NA	9
Liu 2020 (36)	2008–2014	China	Prospective cohort	COPD	275	243	32	70.8	23.2	SII	NA	8
Liu 2020 (36)	2008–2014	China	Prospective cohort	COPD	275	243	32	70.8	23.2	SIRI	NA	8

### 3.2 Study quality assessment

The methodological quality of all included studies was assessed using the NOS. All studies achieved NOS scores ranging from 7 to 9, indicating generally high methodological quality. Because the NOS was originally developed for cohort and case–control studies, its application to cross-sectional studies has certain inherent limitations. To enhance transparency, the scores for individual items, domain-specific subscores, and total NOS scores for each included study are presented in the Supplementary Table S2.

### 3.3 Meta-analysis results

#### 3.3.1 Association between SII and COPD

##### 3.3.1.1 SII and COPD prevalence (categorical analysis)

**Figure 2:**
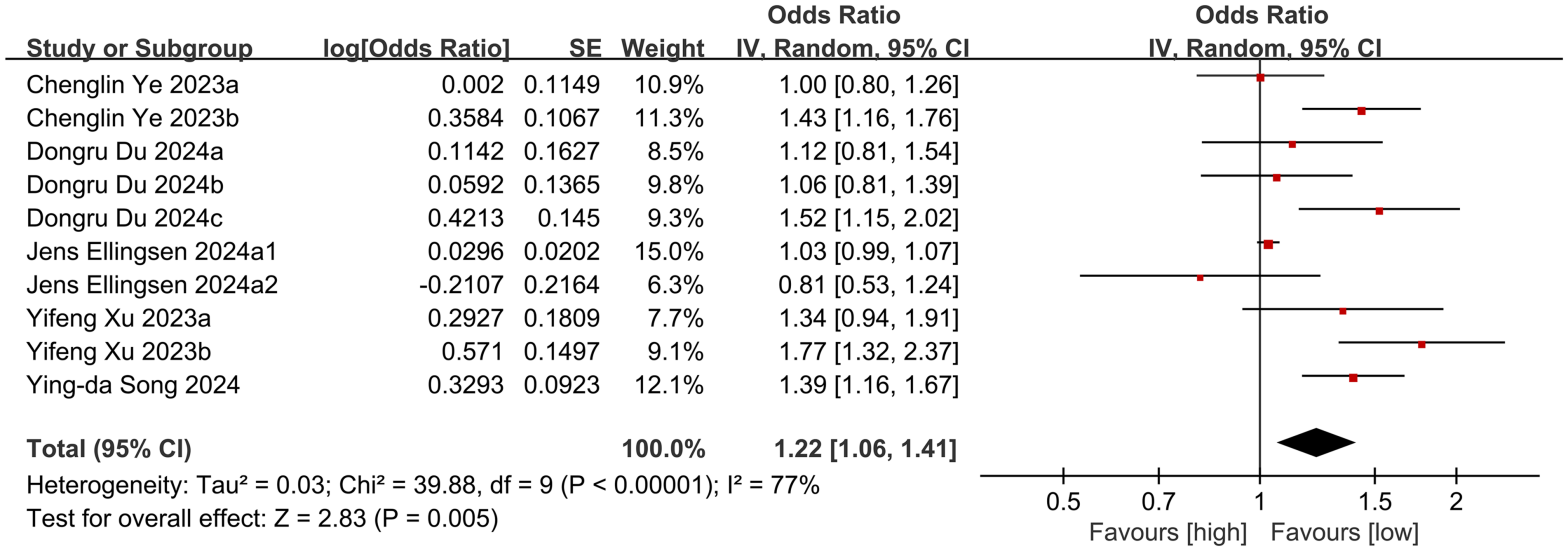
Forest plot of the association between SII and the risk of COPD (categorical).

Ten studies investigated the association between SII and COPD prevalence. Substantial heterogeneity was observed among the included studies (*I*^2^ = 77%, *p <* 0.00001), so a REM was applied (Figure 2). The pooled analysis demonstrated that elevated SII levels were significantly associated with a higher prevalence of COPD (OR = 1.22, 95% CI: 1.06–1.41; *p* = 0.005). To explore potential sources of heterogeneity, subgroup analyses were performed according to study design, age, geographic region, and disease status. The results showed that the association between SII and COPD prevalence was no longer statistically significant in cohort studies, populations aged ≥65, studies conducted in Europe, and patients with acute exacerbations of COPD (AECOPD). In contrast, significant associations remained evident across the other subgroups (Table 2). These findings suggest that study design, age distribution, geographic region, and COPD status were major contributors to the observed heterogeneity. Notably, none of the included studies specifically focused on patients with tuberculosis-associated obstructive pulmonary disease (TOPD) or COPD complicated by COVID-19 infection. Consequently, subgroup analyses stratified by COPD etiology could not be performed.

**Table 2:** Subgroup analysis of SII and the prevalence rate of COPD.

Subgroup	SII and the prevalence rate of COPD(classification)			
	Study	OR [95% CI]	*p*	*I* ^2^
Total	10	1.22 [1.06–1.41]	0.005	77%
Study design				
Cohort	2	1.01 [0.87, 1.16]	0.93	18%
Case–control	8	1.31 [1.14, 1.49]	<0.0001	53%
Mean/median age				
≥65 y	2	1.01 [0.87, 1.16]	0.93	18%
<65 y	3	1.47 [1.26, 1.70]	<0.0001	8%
Region				
Europe	2	1.01 [0.87, 1.16]	0.93	18%
America	8	1.31 [1.14, 1.49]	<0.0001	53%
Disease status				
AECOPD	2	1.01 [0.87, 1.16]	0.93	18%
COPD	8	1.31 [1.14, 1.49]	<0.0001	53%

##### 3.3.1.2 SII and COPD prevalence: categorical analysis (duplicate NHANES data excluded)

**Figure 3:**
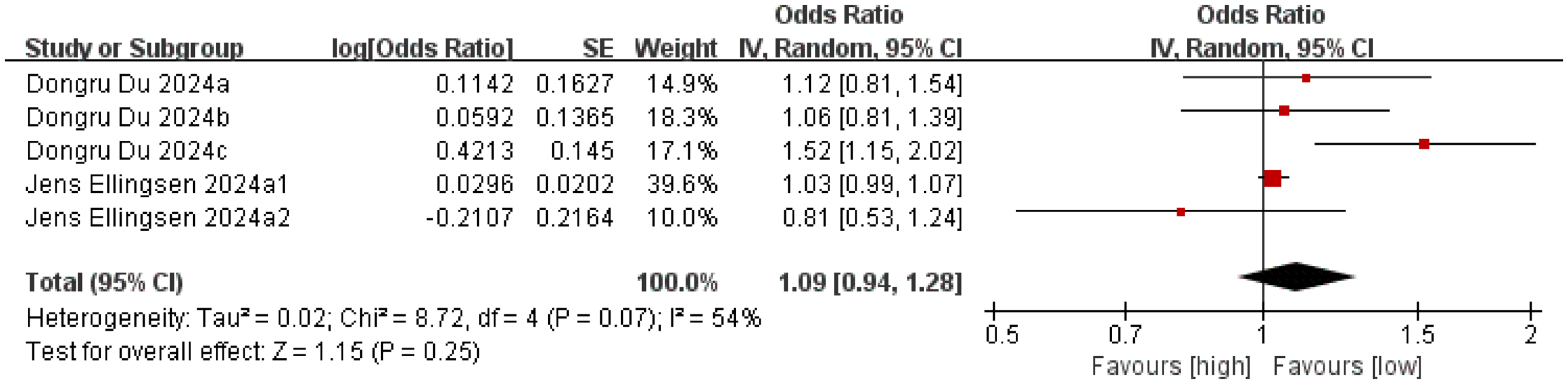
Forest plot of the association between SII and the risk of COPD (categorical; duplicate NHANES data excluded).

Among the included studies, four were derived from the NHANES database and contributed eight comparison groups. Given the potential overlap in study populations across different survey cycles, a sensitivity analysis was conducted by retaining only the NHANES study with the largest sample size and the most comprehensively adjusted model. Following the exclusion of potentially overlapping NHANES datasets, five studies remained eligible for analysis. Moderate heterogeneity was observed (*I*^2^ = 54%, *p* = 0.07), and a REM was employed (Figure 3). The pooled analysis yielded an OR of 1.09 (95% CI: 0.94–1.28; *p* = 0.25), indicating that the association between elevated SII levels and COPD prevalence was no longer statistically significant after accounting for potential population overlap.

##### 3.3.1.3 SII and COPD prevalence (continuous analysis)

**Figure 4:**
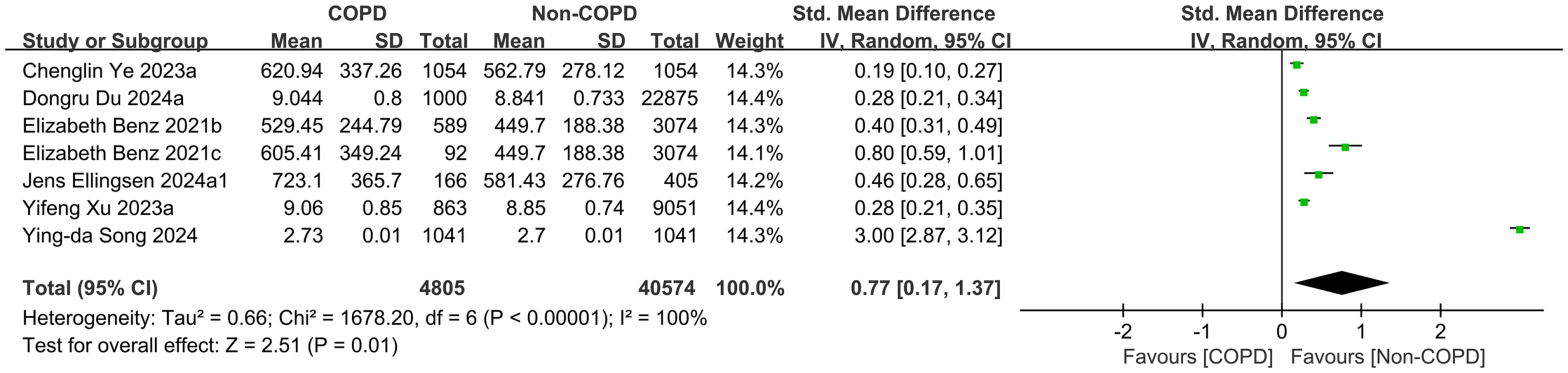
Forest plot of the association between SII and the risk of COPD (continuous).

The association between SII and COPD prevalence was further examined using continuous data from seven studies. Owing to substantial heterogeneity (*I*^2^ = 100%, *p <* 0.00001), a REM was used (Figure 4). The pooled results demonstrated that SII levels were significantly higher in patients with COPD than in healthy controls (SMD = 0.77, 95% CI: 0.17–1.37; *p* = 0.01).

##### 3.3.1.4 SII and ACM (Categorical Analysis)

**Figure 5:**
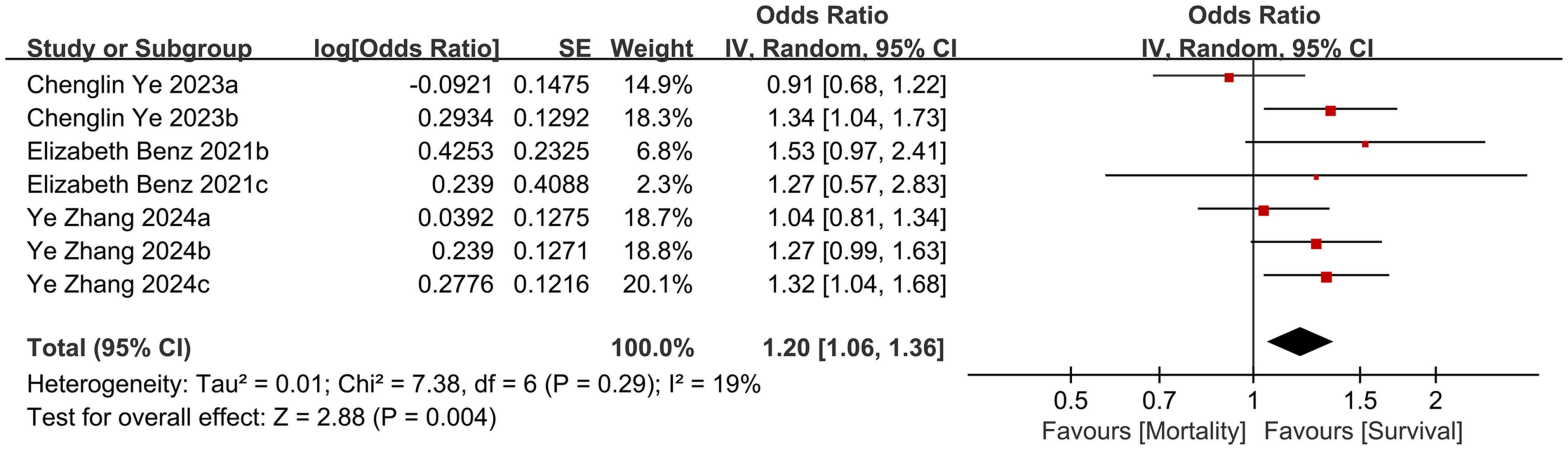
Forest plot of the association between SII and the risk of all-cause mortality in COPD (categorical).

Seven studies assessed the association between SII and ACM. Although heterogeneity was low (*I*^2^ = 19%, *p* = 0.29), a REM was applied in accordance with the predefined analytical strategy (Figure 5). Elevated SII levels were significantly associated with an higher risk of ACM (OR = 1.20, 95% CI: 1.09–1.36; *p* = 0.004).

##### 3.3.1.5 SII and RF in COPD (Categorical Analysis)

**Figure 6:**
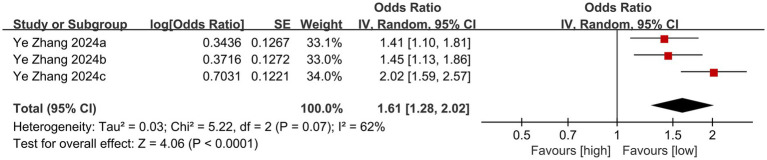
Forest plot of the association between SII and the risk of respiratory failure in COPD (categorical).

Three studies evaluated the relationship between SII and RF among patients with COPD. Moderate heterogeneity was detected (*I*^2^ = 62%, *p* = 0.07), and a REM was therefore used (Figure 6). The pooled analysis indicated that elevated SII levels were significantly associated with an higher risk of RF (OR = 1.61, 95% CI: 1.28–2.02; *p <* 0.0001).

#### 3.3.2 Association between SIRI and COPD

##### 3.3.2.1 SIRI and COPD prevalence (Categorical Analysis)

**Figure 7:**
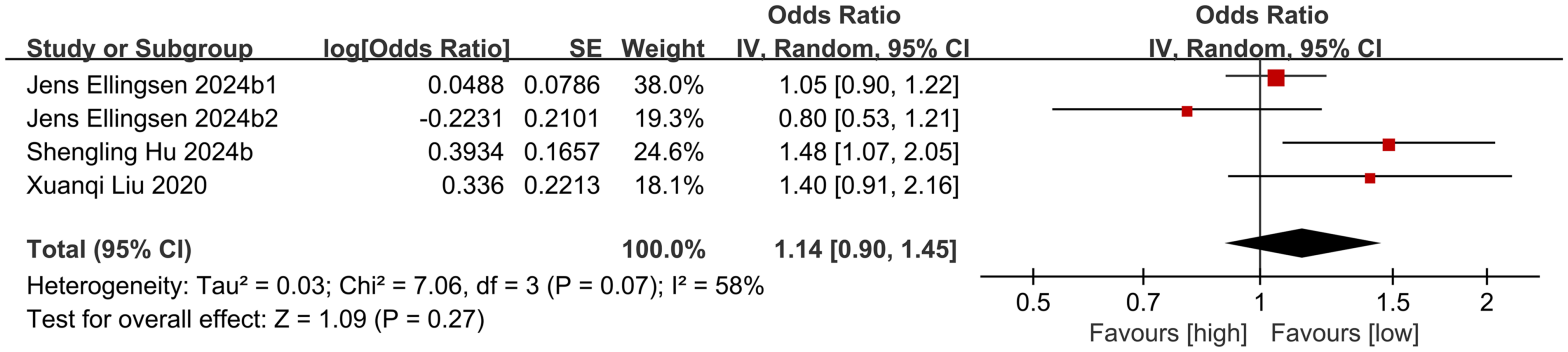
Forest plot of the association between SIRI and the risk of COPD (categorical).

Four studies investigated the association between SIRI and COPD prevalence. Given the presence of moderate heterogeneity (*I*^2^ = 58%, *p* = 0.07), a REM was employed (Figure 7). The pooled analysis demonstrated that higher SIRI levels were not significantly associated with COPD prevalence (OR = 1.14, 95% CI: 0.90–1.45; *p* = 0.27).

### 3.4 Sensitivity analysis

**Figure 8:**
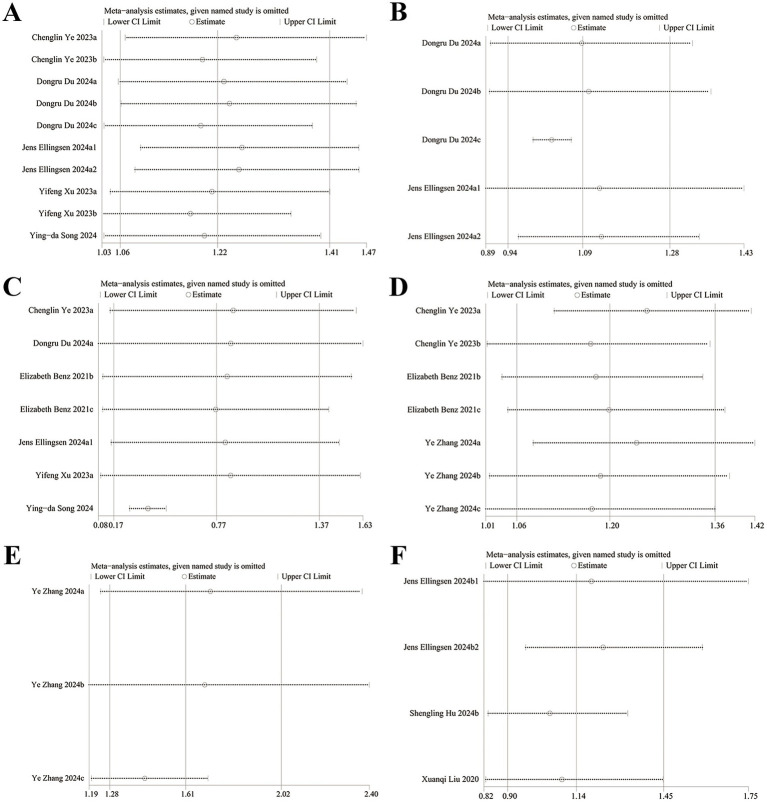
Sensitivity analysis: **(A)** SII and the risk of COPD (categorical); **(B)** SII and the risk of COPD (categorical; duplicate NHANES data excluded); **(C)**SII and the risk of COPD (continuous); **(D)** SII and the risk of all-cause mortality in COPD (categorical); **(E)** SII and the risk of respiratory failure in COPD (categorical); **(F)** SIRI and the risk of COPD (categorical).

Sensitivity analyses were performed to evaluate the robustness of the pooled estimates. For all investigated outcomes, including SII and COPD prevalence (categorical; Figure 8A), SII and COPD Prevalence: (categorical;duplicate NHANES data excluded; Figure 8B), SII and COPD prevalence (continuous; Figure 8C), SII and ACM (categorical;Figure 8D), SII and RF (categorical; Figure 8E), and SIRI and COPD prevalence (categorical; Figure 8F), sequential omission of individual studies did not materially alter the overall effect estimates. The pooled results consistently remained within the corresponding 95% CIs of the primary analyses. These findings indicate that the results were robust and not unduly influenced by any single study.

### 3.5 Publication bias

**Figure 9:**
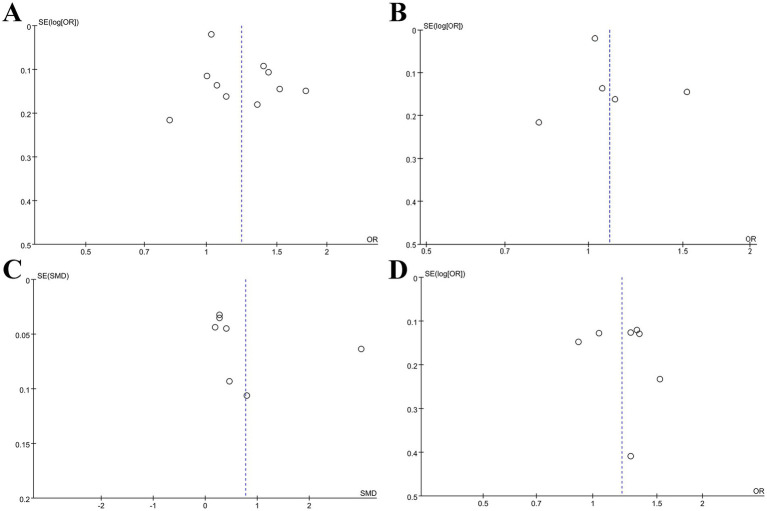
Funnel plot: **(A)** SII and the risk of COPD (categorical); **(B)** SII and the risk of COPD (categorical; duplicate NHANES data excluded); **(C)** SII and the risk of COPD (continuous); **(D)** SII and all-cause mortality in COPD patients (categorical).

Publication bias was evaluated using funnel plots and Egger’s regression test. No significant publication bias was detected for the analyses of SII and COPD prevalence (categorical; *p* = 0.058; Figure 9A), SII and COPD prevalence after exclusion of duplicate NHANES data (categorical; *p* = 0.551; Figure 9B), SII and COPD prevalence (continuous; *p* = 0.310; Figure 9C), SII and ACM (categorical; *p* = 0.779; Figure 9D).

## 4 Discussion

This systematic review and meta-analysis elucidated the associations of SII and SIRI with the prevalence and prognosis of COPD. Based on 11 high-quality studies involving 59,621 participants, several important findings emerged. SII appears to be a robust indicator of COPD prevalence and adverse outcomes: elevated SII levels were associated with the presence of COPD onset (OR = 1.22). Furthermore, among COPD patients, higher SII values were predictive of increased risks of ACM (OR = 1.20) and RF (OR = 1.61). In contrast, the association between SIRI and COPD prevalence did not reach statistical significance, as current evidence did not support a meaningful relationship between elevated SIRI levels and COPD risk (OR = 1.14, *p* = 0.27).

Subgroup analyses identified several important sources of heterogeneity. Specifically, the association between SII and COPD prevalence was not statistically significant in cohort studies, populations aged ≥65, studies conducted in Europe, and patients with acute exacerbations of COPD (AECOPD). These findings suggest that residual confounding may be more influential in prospective cohort studies, that the high burden of comorbidities in older populations may reduce the specificity of SII, and that differences in genetic background, environmental exposures, and healthcare systems may attenuate its predictive value in European populations. Nevertheless, SII remained significantly associated with COPD prevalence across most other subgroups. These observations indicate that the clinical utility of SII may vary according to study design, population characteristics, and geographic setting. Future research should focus on establishing standardized measurement protocols and validating the biomarker across diverse populations.

In addition, the COPD populations included in the present meta-analysis exhibited considerable etiological diversity. Some studies enrolled patients with TOPD, whereas others included individuals with concurrent COVID-19 infection. Chronic *Mycobacterium tuberculosis* infection induces persistent granulomatous inflammation, while COVID-19 is characterized by marked systemic immune activation and cytokine release. Both conditions may independently elevate inflammatory biomarkers, including SII and SIRI. Consequently, the observed associations between elevated SII and adverse outcomes in these populations may partially reflect the inflammatory burden attributable to underlying infectious diseases rather than COPD itself. Such etiological heterogeneity may represent an important contributor to the substantial between-study heterogeneity observed in this meta-analysis. Future studies should therefore adopt more homogeneous definitions of COPD etiology and infectious status to obtain more precise estimates of biomarker performance.

The observed heterogeneity may also be attributable to several methodological and clinical factors. First, the included studies employed diverse designs, including prospective cohort studies, retrospective case–control studies, and analyses of electronic health record databases, each with distinct capacities for confounding control and different risks of bias. Second, substantial variability existed in study populations, COPD diagnostic criteria, and disease severity, with participants ranging from community-based individuals to critically ill hospitalized patients. Third, the timing of SII assessment and the cutoff values used for risk stratification varied considerably across studies. In addition, adjustment for important confounders such as age, smoking status, and comorbidities was inconsistent. Therefore, the pooled estimates reported herein should be interpreted as approximations of the underlying effect rather than universally applicable effect sizes. These findings underscore the need for future studies to standardize biomarker measurement protocols, improve population homogeneity, and implement comprehensive confounder adjustment strategies.

The associations of SII and SIRI with COPD identified in this meta-analysis are in line with prior evidence concerning immune-inflammatory markers in COPD populations. A meta-analysis published in 2021 by Angelo Zinellu et al. (37), including 27 studies with approximately 14,000 participants, reported significantly higher PCs and platelet-to-lymphocyte ratio (PLR) values in patients with stable COPD compared with healthy controls, with even greater elevations observed during acute exacerbations. These findings support the utility of PLR as a marker of chronic inflammation and acute disease worsening. More recently, a meta-analysis conducted by Li Fang, Jianzhi Zhu, and Dandan Fu (38), incorporating 24 studies and more than 18,600 patients with COPD, demonstrated that NLR was independently associated with increased ACM risk, particularly during acute exacerbations. Consistent with these observations, the present study further demonstrates that SII, as a composite inflammatory biomarker integrating multiple immune cell components, is significantly associated with COPD prevalence, ACM, and RF, thereby expanding the current evidence base supporting the prognostic value of systemic inflammatory markers in COPD.

SII is an emerging composite inflammatory biomarker, and its association with COPD has attracted increasing attention (22, 39). The biological basis underlying this association is closely linked to chronic systemic inflammation, a hallmark pathophysiological feature of COPD (19, 40). Several mechanisms may account for this association. First, neutrophil-driven inflammation and tissue destruction play central roles in COPD pathogenesis. Airway inflammation in COPD is characterized by predominant neutrophilic infiltration (41, 42). Neutrophils, which constitute a key component of both SII and SIRI, directly reflect the activation of innate immune responses and systemic inflammatory status (43, 44). Activated neutrophils release a variety of proteolytic enzymes, including neutrophil elastase and MMPs, as well as reactive oxygen species (ROS), thereby contributing to alveolar wall destruction, emphysematous changes, and airway remodeling (45–47). Second, immune dysregulation and lymphocyte depletion may further promote disease progression. COPD is frequently accompanied by impaired cellular immune function and altered adaptive immune responses (48, 49). Elevated PCs, reflected in the numerator of SII, indicate a state of platelet-mediated thrombo-inflammation (50). Activated platelets exacerbate endothelial dysfunction and pulmonary vascular remodeling through the release of inflammatory mediators and interactions with leukocytes (51, 52). By integrating information from neutrophils, lymphocytes, and platelets, SII may therefore provide a more comprehensive representation of the overall inflammatory burden and immune dysregulation present in patients with COPD than single-cell-derived inflammatory markers (53–56). Elevated SII levels are significantly associated with an increased risk of acute exacerbations, greater disease severity, and higher ACM in COPD. Moreover, SII often demonstrates superior predictive performance compared with individual hematological indices. Therefore, SII represents a highly promising tool for prognostic assessment in COPD.

The present study demonstrated associations between elevated SII levels and COPD prevalence, ACM, and RF, thereby providing preliminary evidence supporting its potential clinical applicability. From a practical perspective, this readily available and inexpensive biomarker may serve as a useful adjunct in several clinical settings. In outpatient clinics and community-based screening programs, elevated SII levels may alert clinicians to individuals with a history of smoking or respiratory symptoms who warrant closer evaluation and prioritization for pulmonary function testing. Among patients hospitalized with acute exacerbations of COPD, baseline or serial measurements of SII may facilitate early identification of those at increased risk of RF or mortality, thereby supporting enhanced monitoring and timely optimization of therapeutic strategies. In addition, SII may help quantify systemic inflammatory burden and provide an objective indicator for integrated, inflammation-oriented management approaches. Nevertheless, current evidence supports the use of SII as a complementary component of a comprehensive risk assessment framework rather than as a standalone diagnostic or prognostic tool. Its clinical implementation will require the establishment of standardized cutoff values and validation in prospective interventional studies to confirm its capacity to improve clinical outcomes.

As an easily accessible composite inflammatory biomarker, SII is correlated with COPD prevalence, ACM, and RF, and demonstrates better prognostic performance than SIRI. Its limited effect size suggests that SII should be regarded as an adjunctive biomarker within a comprehensive risk assessment framework rather than a substitute for established prognostic indicators, such as pulmonary function parameters, GOLD classification, exacerbation history, and the BODE index. Furthermore, the substantial etiological heterogeneity across the included studies represents an important limitation of the present analysis and may partially restrict the generalizability of the pooled findings to patients with typical COPD who do not have concomitant infectious conditions. Future studies are needed to determine whether incorporating SII into existing prognostic models provides meaningful incremental predictive value, as reflected by improvements in discrimination, risk reclassification, and clinical utility, including the C-statistic, net reclassification improvement (NRI), and decision-curve analysis. In addition, stratified investigations across different COPD etiologies and disease states, including stable disease and acute exacerbations, shall further define the clinical applicability and prognostic utility of SII.

## 5 Conclusion

Higher SII levels are associated with a higher prevalence of COPD and higher risks of ACM and RF among patients with COPD. In contrast, the association between SIRI and COPD prevalence was not statistically significant. These findings suggest that SII may represent a promising and readily accessible biomarker for risk stratification and prognostic assessment in COPD. However, several limitations should be acknowledged. Most included studies were retrospective in nature and therefore susceptible to inherent biases. In addition, geographic selection bias, substantial between-study heterogeneity, and the relatively modest effect sizes observed may limit the robustness and generalizability of the findings. Consequently, large-scale, multicenter prospective studies are warranted to establish standardized application criteria, validate optimal cutoff values, and determine whether incorporation of SII into existing clinical prediction models can improve risk stratification and clinical management in patients with COPD.

## Data Availability

The original contributions presented in the study are included in the article/Supplementary material, further inquiries can be directed to the corresponding author/s.

## References

[1] Alabi FO Alkhateeb HA DeBarros KM Barletti Benel PS Sanchez-Martez RL Zeper ML . The heterogeneity of COPD patients in a community-based practice and the inadequacy of the Global Initiative for Chronic Obstructive Lung Disease criteria: a real-world experience. Chron Obstruct Pulm Dis. (2021) 8:396–407. doi: 10.15326/jcopdf.2021.0229, 34236778 PMC8428596

[2] Rycroft CE Heyes A Lanza L Becker K. Epidemiology of chronic obstructive pulmonary disease: a literature review. Int J Chron Obstruct Pulmon Dis. (2012) 7:457–94. doi: 10.2147/copd.S32330, 22927753 PMC3422122

[3] Amaral AFS Burney PGJ Patel J Minelli C Mejza F Mannino DM . Chronic airflow obstruction and ambient particulate air pollution. Thorax. (2021) 76:1236–41. doi: 10.1136/thoraxjnl-2020-216223, 33975927 PMC8606424

[4] Holtjer JCS Bloemsma LD Beijers R Cornelissen MEB Hilvering B Houweling L . Identifying risk factors for COPD and adult-onset asthma: an umbrella review. Eur Respir Rev. (2023) 32:230009. doi: 10.1183/16000617.0009-2023, 37137510 PMC10155046

[5] McIvor RA Tunks M Todd DC. COPD. BMJ Clin Evid. (2011) 2011:1502. Available online at: https://pmc.ncbi.nlm.nih.gov/articles/PMC2907933/PMC3275305 21639960

[6] Gueçamburu M Zysman M. Place des biothérapies dans la BPCO [Biologic agents in COPD management]. Rev Mal Respir. (2024) 41:127–38. doi: 10.1016/j.rmr.2023.11.003 38129268

[7] Gené RJ Giugno ER Abbate EH Figueroa-Casas JC Mazzei JA Schiavi EA. Updated Argentine consensus on chronic obstructive pulmonary disease. Medicina. (2003) 63:419–46. Available online at: https://europepmc.org/article/MED/14628655 14628655

[8] Riley CM Sciurba FC. Diagnosis and Outpatient Management of Chronic Obstructive Pulmonary Disease: A Review. JAMA. (2019) 321:786–97. doi: 10.1001/jama.2019.0131, 30806700

[9] Tantucci C Modina D. Lung function decline in COPD. Int J Chron Obstruct Pulmon Dis. (2012) 7:95–9. doi: 10.2147/copd.S27480, 22371650 PMC3282601

[10] Vitacca M. Exacerbations of COPD: predictive factors, treatment and outcome. Monaldi Arch Chest Dis. (2001) 56:137–43. Available online at: https://europepmc.org/article/MED/11499303 11499303

[11] Hartl S Lopez-Campos JL Pozo-Rodriguez F Castro-Acosta A Studnicka M Kaiser B . Risk of death and readmission of hospital-admitted COPD exacerbations: European COPD Audit. Eur Respir J. (2016) 47:113–21. doi: 10.1183/13993003.01391-2014, 26493806

[12] Shrikrishna D Steer J Bostock B Dickinson SW Piwko A Ramalingam S . Chronic Obstructive Pulmonary Disease and the management of cardiopulmonary risk in the UK: a systematic literature review and modified delphi study. Int J Chron Obstruct Pulmon Dis. (2025) 20:2073–90. doi: 10.2147/copd.S523865, 40585423 PMC12206411

[13] Brandsma CA Van den Berge M Hackett TL Brusselle G Timens W. Recent advances in chronic obstructive pulmonary disease pathogenesis: from disease mechanisms to precision medicine. J Pathol. (2020) 250:624–35. doi: 10.1002/path.5364, 31691283 PMC7216938

[14] Maniscalco M Candia C Ambrosino P Iovine A Fuschillo S. Chronic obstructive pulmonary disease's eosinophilic phenotype: Clinical characteristics, biomarkers and biotherapy. Eur J Intern Med. (2025) 131:27–35. doi: 10.1016/j.ejim.2024.10.015, 39443246

[15] Hogea SP Tudorache E Fildan AP Fira-Mladinescu O Marc M Oancea C. Risk factors of chronic obstructive pulmonary disease exacerbations. Clin Respir J. (2020) 14:183–97. doi: 10.1111/crj.13129, 31814260

[16] Barnes PJ Burney PG Silverman EK Celli BR Vestbo J Wedzicha JA . Chronic obstructive pulmonary disease. Nat Rev Dis Primers. (2015) 1:15076. doi: 10.1038/nrdp.2015.76, 27189863

[17] Yayan J Rasche K. Asthma and COPD: Similarities and Differences in the Pathophysiology, Diagnosis and Therapy. Adv Exp Med Biol. (2016) 910:31–8. doi: 10.1007/5584_2015_206, 26820733

[18] Jeffery PK. Comparison of the structural and inflammatory features of COPD and asthma. Giles F. Filley Lecture. Chest. (2000) 117:251s–60s. doi: 10.1378/chest.117.5_suppl_1.251s 10843939

[19] Barnes PJ. Inflammatory mechanisms in patients with chronic obstructive pulmonary disease. J Allergy Clin Immunol. (2016) 138:16–27. doi: 10.1016/j.jaci.2016.05.011, 27373322

[20] Barnes PJ Celli BR. Systemic manifestations and comorbidities of COPD. Eur Respir J. (2009) 33:1165–85. doi: 10.1183/09031936.00128008, 19407051

[21] Zhang Y Tan X Hu S Cui Z Chen W. Relationship between systemic immune-inflammation index and risk of respiratory failure and death in COPD: a retrospective cohort study based on the MIMIC-IV Database. Int J Chron Obstruct Pulmon Dis. (2024) 19:459–73. doi: 10.2147/copd.S446364, 38404653 PMC10888109

[22] Ye C Yuan L Wu K Shen B Zhu C. Association between systemic immune-inflammation index and chronic obstructive pulmonary disease: a population-based study. BMC Pulm Med. (2023) 23:295. doi: 10.1186/s12890-023-02583-5, 37563621 PMC10416535

[23] Page MJ McKenzie JE Bossuyt PM Boutron I Hoffmann TC Mulrow CD . The PRISMA 2020 statement: an updated guideline for reporting systematic reviews. BMJ. (2021) 372:n71. doi: 10.1136/bmj.n71, 33782057 PMC8005924

[24] Ren Q Li Y Chen H Chen Y. Prognostic value of lymphocyte to monocyte ratio for the patients with bladder cancer: a systematic review and meta-analysis. Front Oncol. (2025) 15:1601040. doi: 10.3389/fonc.2025.1601040, 41114376 PMC12532007

[25] Kim SR Kim K Lee SA Kwon SO Lee JK Keum N . Effect of red, processed, and white meat consumption on the risk of gastric cancer: an overall and dose-response meta-analysis. Nutrients. (2019) 11. doi: 10.3390/nu11040826, 30979076 PMC6520977

[26] Higgins JP Thompson SG. Quantifying heterogeneity in a meta-analysis. Stat Med. (2002) 21:1539–58. doi: 10.1002/sim.1186, 12111919

[27] Egger M Davey Smith G Schneider M Minder C. Bias in meta-analysis detected by a simple, graphical test. BMJ. (1997) 315:629–34. doi: 10.1136/bmj.315.7109.629, 9310563 PMC2127453

[28] Benz E Wijnant SRA Trajanoska K Arinze JT de Roos EW de Ridder M . Sarcopenia, systemic immune-inflammation index and all-cause mortality in middle-aged and older people with COPD and asthma: a population-based study. ERJ Open Res. (2022) 8:00628–2021. doi: 10.1183/23120541.00628-2021, 35036418 PMC8752940

[29] Hu S Yu Q Liu F Gong F. A novel inflammatory indicator for tuberculosis-associated obstructive pulmonary disease (TOPD): the systemic inflammatory response index (SIRI). J Inflamm Res. (2024) 17:4219–28. doi: 10.2147/jir.S468232, 38974002 PMC11227324

[30] Hosseninia S Ghobadi H Garjani K Hosseini SAH Aslani MR. Aggregate index of systemic inflammation (AISI) in admission as a reliable predictor of mortality in COPD patients with COVID-19. BMC Pulm Med. (2023) 23:107. doi: 10.1186/s12890-023-02397-5, 37003999 PMC10063934

[31] Zuo H Xie X Peng J Wang L Zhu R. Predictive value of novel inflammation-based biomarkers for pulmonary hypertension in the acute exacerbation of chronic obstructive pulmonary disease. Anal Cell Pathol. (2019) 2019:1–9. doi: 10.1155/2019/5189165, 31737467 PMC6815641

[32] Ellingsen J Janson C Bröms K Hårdstedt M Högman M Lisspers K . CRP, fibrinogen, white blood cells, and blood cell indices as prognostic biomarkers of future COPD exacerbation frequency: the TIE cohort study. J Clin Med. (2024) 13. doi: 10.3390/jcm13133855, 38999421 PMC11242174

[33] Xu Y Yan Z Li K Liu L. The association between systemic immune-inflammation index and chronic obstructive pulmonary disease in adults aged 40 years and above in the United States: a cross-sectional study based on the NHANES 2013-2020. Front Med. (2023) 10:1270368. doi: 10.3389/fmed.2023.1270368, 38076255 PMC10704483

[34] Song YD Bai XM Ma J. The association of systemic immune-inflammation index with lung function, risk of COPD and COPD severity: A population-based study. PLoS One. (2024) 19:e0303286. doi: 10.1371/journal.pone.0303286, 38875233 PMC11178193

[35] Du D Zhang G Xu D Liu L Hu X Zeng T . Association between systemic inflammatory markers and chronic obstructive pulmonary disease: a population-based study. Heliyon. (2024) 10:e31524. doi: 10.1016/j.heliyon.2024.e31524, 38818179 PMC11137537

[36] Liu X Ge H Feng X Hang J Zhang F Jin X . The Combination of Hemogram Indexes to Predict Exacerbation in Stable Chronic Obstructive Pulmonary Disease. Front Med. (2020) 7:572435. doi: 10.3389/fmed.2020.572435, 33381510 PMC7769039

[37] Zinellu A Paliogiannis P Sotgiu E Mellino S Fois AG Carru C . Platelet count and platelet indices in patients with stable and acute exacerbation of chronic obstructive pulmonary disease: a systematic review and meta-analysis. COPD. (2021) 18:231–45. doi: 10.1080/15412555.2021.1898578, 33929925

[38] Fang L Zhu J Fu D. Predictive value of neutrophil-lymphocyte ratio for all-cause mortality in patients with chronic obstructive pulmonary disease: a systematic review and meta-analysis. BMC Pulm Med. (2025) 25:206. doi: 10.1186/s12890-025-03677-y, 40301774 PMC12039089

[39] Fu Y Wang Y Wang Y Mou T He X Wang J . Biomarkers (NLR, PLR, SII) for Frequent COPD Exacerbations: Diagnostic and Clinical Management Implications in a Retrospective Study. Int J Chron Obstruct Pulmon Dis. (2025) 20:987–98. doi: 10.2147/copd.S510118, 40207023 PMC11980941

[40] Xu J Zeng Q Li S Su Q Fan H. Inflammation mechanism and research progress of COPD. Front Immunol. (2024) 15:1404615. doi: 10.3389/fimmu.2024.1404615, 39185405 PMC11341368

[41] Lange P Ahmed E Lahmar ZM Martinez FJ Bourdin A. Natural history and mechanisms of COPD. Respirology. (2021) 26:298–321. doi: 10.1111/resp.14007, 33506971

[42] Wang Y Xu J Meng Y Adcock IM Yao X. Role of inflammatory cells in airway remodeling in COPD. Int J Chron Obstruct Pulmon Dis. (2018) 13:3341–8. doi: 10.2147/copd.S176122, 30349237 PMC6190811

[43] Wang RH Wen WX Jiang ZP Du ZP Ma ZH Lu AL . The clinical value of neutrophil-to-lymphocyte ratio (NLR), systemic immune-inflammation index (SII), platelet-to-lymphocyte ratio (PLR) and systemic inflammation response index (SIRI) for predicting the occurrence and severity of pneumonia in patients with intracerebral hemorrhage. Front Immunol. (2023) 14:1115031. doi: 10.3389/fimmu.2023.1115031, 36860868 PMC9969881

[44] Islam MM Satici MO Eroglu SE. Unraveling the clinical significance and prognostic value of the neutrophil-to-lymphocyte ratio, platelet-to-lymphocyte ratio, systemic immune-inflammation index, systemic inflammation response index, and delta neutrophil index: An extensive literature review. Turk J Emerg Med. (2024) 24:8–19. doi: 10.4103/tjem.tjem_198_23, 38343523 PMC10852137

[45] Rodrigues SF Granger DN. Blood cells and endothelial barrier function. Tissue Barriers. (2015) 3:e978720. doi: 10.4161/21688370.2014.978720, 25838983 PMC4372023

[46] Li H Cui D Tong X Ma N Gao Y Cui X . The role of matrix metalloproteinases in extracellular matrix remodelling in chronic obstructive pulmonary disease rat models. Zhonghua Nei Ke Za Zhi. (2002) 41:393–8. Available online at: https://rs.yiigle.com/cmaid/1142301 12137602

[47] Burgel PR Bourdin A Pilette C Garcia G Chanez P Tillie-Leblond I. Structural abnormalities and inflammation in COPD: a focus on small airways. Rev Mal Respir. (2011) 28:749–60. doi: 10.1016/j.rmr.2011.01.009, 21742236

[48] Polverino F Sin DD. Type 2 airway inflammation in COPD. Eur Respir J. (2024) 63:2400150. doi: 10.1183/13993003.00150-2024 38485148

[49] Zhang F Cui Y Zhang T Yin W. Epigenetic regulation of macrophage activation in chronic obstructive pulmonary disease. Front Immunol. (2024) 15:1445372. doi: 10.3389/fimmu.2024.1445372, 39206196 PMC11349576

[50] Desai C Koupenova M Machlus KR Sen Gupta A. Beyond the thrombus: Platelet-inspired nanomedicine approaches in inflammation, immune response, and cancer. J Thromb Haemost. (2022) 20:1523–34. doi: 10.1111/jth.15733, 35441793 PMC9321119

[51] Marques P Bocigas I Domingo E Francisco V Tarraso J Garcia-Sanjuan Y . Key role of activated platelets in the enhanced adhesion of circulating leucocyte-platelet aggregates to the dysfunctional endothelium in early-stage COPD. Front Immunol. (2024) 15:1441637. doi: 10.3389/fimmu.2024.1441637, 39229275 PMC11369892

[52] Liang LS Mo Y Zhang ZY Liang PS Xu P. Progress in platelets and chronic obstructive pulmonary disease. Zhonghua Jie He He Hu Xi Za Zhi. (2022) 45:1050–4. doi: 10.3760/cma.j.cn112147-20220425-00349, 36207962

[53] Zhou K Wen Q Zuo Y Bai G Sun R. Pathogenic Cell in COPD: Mechanisms of Airway Remodeling, Immune Dysregulation, and Therapeutic Implications. Int J Chron Obstruct Pulmon Dis. (2025) 20:2925–43. doi: 10.2147/copd.S523519, 40860610 PMC12377387

[54] Pang X Liu X. Immune dysregulation in chronic obstructive pulmonary disease. Immunol Investig. (2024) 53:652–94. doi: 10.1080/08820139.2024.2334296, 38573590

[55] Balbi B Sangiorgi C Gnemmi I Ferrarotti I Vallese D Paracchini E . Bacterial load and inflammatory response in sputum of alpha-1 antitrypsin deficiency patients with COPD. Int J Chron Obstruct Pulmon Dis. (2019) 14:1879–93. doi: 10.2147/copd.S207203, 31686800 PMC6709647

[56] Beech AS Lea S Kolsum U Wang Z Miller BE Donaldson GC . Bacteria and sputum inflammatory cell counts; a COPD cohort analysis. Respir Res. (2020) 21:289. doi: 10.1186/s12931-020-01552-4, 33131502 PMC7603729

