# Supplementary material for: Associations of the systemic immune-inflammation index and systemic inflammatory response index with chronic obstructive pulmonary disease: a systematic review and meta-analysis
Source: Front Med (Lausanne). 2026 Jun 26;13:1853538. doi: 10.3389/fmed.2026.1853538 (PMC13350035; doi:10.3389/fmed.2026.1853538)
Supplement: Supplementary file 1 [file Table_1.DOCX]

**Table S1.** The Newcastle-Ottawa Scale was used to assess the quality of the case-control studies that met the criteria.

| Study | Selection | | | | Comparability | | Exposure factors | | |
| --- | --- | --- | --- | --- | --- | --- | --- | --- | --- |
|  | Appropriate case | Case representativeness | Comtrol selection | Determination of contrast | Comparability on most important factors | Comparability on other risk factors | Identification of exposure factors | Method to determine exposure factors | No response |
| Ye 2023 | * | * | * | * | * | - | * | - | * |
| Hu 2024 | * | * | * | * | - | - | * | * | * |
| Hosseninia 2023 | * | * | * | - | * | - | * | * | * |
| Zuo 2019 | * | * | * | * | - | - | * | * | * |
| Zhang 2024 | * | * | * | * | - | - | * | * | - |
| Xu 2023 | * | * | * | * | * | - | * | - | * |
| Song 2024 | * | * | * | * | * | - | * | * | - |
| Du 2024 | * | * | * | * | - | - | * | * | * |

*indicates criterion met; - indicates significant of criterion not met.

**Table S2.** The Newcastle-Ottawa Scale was used to assess the quality of the cohort studies that met the criteria.

| Study | Selection | | | | Comparability | | Outcome | | |
| --- | --- | --- | --- | --- | --- | --- | --- | --- | --- |
|  | Representative-ness | Selection of  non-exposed | Ascertainment  of exposure | Outcome not present at start | Comparability on most important factors | Comparability on other risk factors | Assessment of outcome | Long enough follow-up (median≥1 year) | Adequacy  (completeness) of follow-up |
| Ellingsen 2024 | * | * | * | * | * | - | * | * | - |
| Elizabeth 2021 | * | * | * | * | * | - | * | * | * |
| Liu 2020 | * | * | - | * | * | - | * | * | * |

*indicates criterion met; - indicates significant of criterion not met.
